# Gut microbiota-mitochondrial crosstalk in obesity: novel mechanistic insights and therapeutic strategies with traditional Chinese medicine

**DOI:** 10.3389/fphar.2025.1574887

**Published:** 2025-04-22

**Authors:** Lingmiao Wen, Kun Yang, Jiexin Wang, Hang Zhou, Weijun Ding

**Affiliations:** School of Basic Medical Sciences, Chengdu University of Traditional Chinese Medicine, Chengdu, China

**Keywords:** gut microbiota, mitochondria, obesity, metabolism, traditional Chinese medicine

## Abstract

Obesity rates are rising globally and have become a major public health issue. Recent research emphasizes the bidirectional communication between gut microbiota and mitochondrial function in obesity development. Gut microbiota regulates energy metabolism through metabolites that impact mitochondrial processes, such as oxidative phosphorylation, biogenesis, and autophagy. In turn, alterations in mitochondrial function impact microbiota homeostasis. Traditional Chinese medicine (TCM), which encompasses TCM formulas and the metabolites of botanical drugs, employs a holistic and integrative approach that shows promise in regulating gut microbiota–mitochondrial crosstalk. This review systematically explores the intricate interactions between gut microbiota and mitochondrial function, underscoring their crosstalk as a critical mechanistic axis in obesity pathogenesis. Furthermore, it highlights the potential of TCM in developing innovative, targeted interventions, paving the way for personalized approaches in obesity treatment through the precise modulation of gut microbiota–mitochondrial interactions, offering more effective and individualized therapeutic options.

## 1 Introduction

The global incidence of obesity, a multifaceted metabolic disorder, has surged in recent decades, becoming a pressing public health challenge (Welsh et al., 2024). While excessive calorie intake and inactivity are known causes of obesity, attention is shifting toward gut microbiota and mitochondrial interactions (Belda et al., 2022). In a healthy state, the gut microbiota supports host energy metabolism, lipid metabolism, and glucose homeostasis through diverse metabolic activities (Fan and Pedersen, 2021). This microbiota supports the intestinal barrier functionality through the modulation of the host’s cellular processes and immune responses (Leshem et al., 2020). However, a disruption in the gut microbial balance can increase intestinal permeability, thereby allowing bacterial toxins and metabolic products to enter the bloodstream and disrupt the body’s overall metabolic equilibrium (Gasmi et al., 2021). Unfortunately, the exact mechanisms within the intestinal microbial community that trigger these metabolic abnormalities remain poorly understood (Michaudel and Sokol, 2020).

Gut microbiota–mitochondria interactions play a crucial role in various health conditions has become increasingly evident (Ballard and Towarnicki, 2020; Li Y. et al., 2023). By metabolizing dietary elements, gut microbiota generates short-chain fatty acids (SCFAs) and secondary bile acids (SBAs), which influence mitochondrial oxidative phosphorylation (OXPHOS), biogenesis, dynamics, and autophagy mechanisms, thereby significantly affecting energy balance and metabolism (Yoo et al., 2021; Fogelson et al., 2023). As the main cellular powerhouses, mitochondria are pivotal in regulating fat metabolism, thermoregulation, and oxidative balance (Brestoff et al., 2021; Xia et al., 2024). Dysfunctional mitochondria can produce excess reactive oxygen species (ROS) and organic acids, subsequently altering the gut microbiota composition and function, leading to intricate two-way interactions (Singh et al., 2022).

The ancient Chinese medical text *Lingshu Jing* of the *Huangdi Neijing* states that obesity is characterized by “people having fat, ointment, and flesh.” It describes obesity as a condition primarily caused by spleen and kidney deficiency and liver qi stagnation, with phlegm, dampness, heat, and blood stasis as its key pathological manifestations. These factors interact with one another, affecting both energy metabolism and material metabolism in the body. Based on this understanding, traditional Chinese medicine (TCM) has been widely applied in the clinical treatment of obesity, demonstrating safe, gentle, and long-lasting effects, and has accumulated extensive experience through long-term practice (Li et al., 2020; Chen Y. K. et al., 2023; Zhang Q. et al., 2023). Rooted in TCM, TCM formulas and metabolites of botanical drugs offer a promising approach to managing obesity by modulating gut microbiota and mitochondrial function. By restoring microbial balance and enhancing mitochondrial efficiency, it addresses key dysfunctions within the gut microbiota–mitochondria axis, providing a novel and integrative approach to targeted obesity interventions.

Although early research has revealed a sophisticated relationship between gut microbiota and mitochondria, the precise mechanisms underlying this interaction in obesity development remain to be fully understood. This review systematically examines the intricate crosstalk between gut microbiota and mitochondria, emphasizing their role as a mechanistic axis in obesity. Furthermore, it explores the potential of TCM-based interventions in modulating this axis, highlighting their prospective applications in precision medicine approaches for obesity treatment.

## 2 Review methodology

This review conducted a comprehensive literature search across PubMed, Web of Science, ScienceDirect, Google Scholar, and CNKI using a combination of controlled vocabulary and free-text keywords, including “gut microbiota,” “mitochondria,” “obesity,” “metabolic disorders,” “plant metabolites,” “botanical drugs,” and “Traditional Chinese Medicine.” Boolean operators (AND, OR) and database-specific filters were applied to refine results and exclude irrelevant publications. Inclusion criteria encompassed studies investigating gut microbiota-mitochondria interactions in obesity and metabolic disorders using rigorously designed *in vivo* or *in vitro* models. Exclusion criteria comprised studies with unclear experimental design, insufficient mechanistic evaluation, inadequate sample sizes, unsupported conclusions, or redundant/duplicate publications.

## 3 Mitochondrial dysfunction in obesity

**FIGURE 1:**
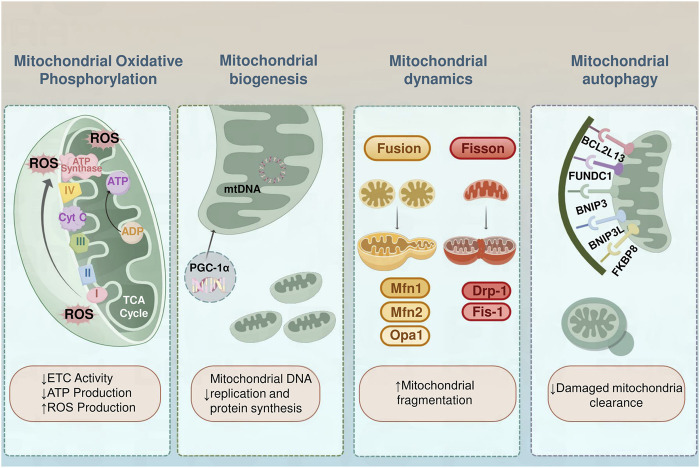
Mitochondrial Dysfunction in Obesity. In individuals with obesity, diminished electron transport chain function results in lower ATP generation and elevated ROS levels, inducing oxidative stress and harm to cells. The process of mitochondrial biogenesis is controlled by PGC-1α, with reduced biogenesis resulting in decreased mitochondrial DNA duplication and protein production. The balance of mitochondrial dynamics is compromised, characterized by enhanced fission facilitated by Drp1 and Fis1, coupled with reduced fusion facilitated by Mfn1/2 and Opa1, leading to a deterioration of network cohesiveness. Mitochondrial autophagy, the process of clearing damaged mitochondria, is impaired, leading to their accumulation. Key mitophagy receptors interacting with the LC3 are essential for the management of mitochondrial quality. BCL2L13 and FUNDC1 bind LC3 to facilitate autophagic clearance, especially under hypoxic conditions. The BNIP3 and BNIP3L possess LC3-interacting region motifs essential for mitochondrial recycling under stressful conditions. FKBP8 also interacts with LC3 to facilitate the clearance of dysfunctional mitochondria.

Mitochondria generate adenosine triphosphate (ATP) through OXPHOS, providing the necessary energy for cellular activities. Beyond energy production, mitochondria are integral to numerous vital cellular functions, including apoptosis control, calcium homeostasis, lipid metabolism, and thermogenesis. Maintaining mitochondrial homeostasis involves balancing and regulating various processes, including OXPHOS, dynamics, biogenesis, and autophagy, all of which are critical for sustaining energy metabolism. Disruptions in these processes can lead to obesity (Figure 1).

### 3.1 Mitochondrial oxidative phosphorylation

Mitochondrial OXPHOS, a key component of cellular respiration, converts chemical energy from nutrients into ATP, the primary energy source for cellular functions (Harrington et al., 2023; Talari et al., 2023). Under normal conditions, electrons move through the electron transport chain sequentially from complexes I to IV, where they ultimately combine with oxygen to form water. As the electrons travel, their movement drives the transfer of protons from the mitochondrial matrix to the intermembrane space. This process creates an electrochemical gradient across the inner mitochondrial membrane. (Pedersen et al., 2022). ATP synthase utilizes this gradient to synthesize ATP from ADP and inorganic phosphate, fueling cellular functions. However, dysfunctional mitochondria impair this process by reducing ATP production and increasing oxidative stress (CA et al., 2021). Obese individuals exhibit significantly reduced activity in complexes I, III, and IV, impairing the effectiveness of the electron transport chain and disrupting ATP production. This dysfunction also increases electron leakage from the chain, leading to the excessive production of ROS (MacLean et al., 2023; Zotta et al., 2024). The accumulation of ROS exacerbates damage to mitochondria and other cell structures, perpetuating a detrimental cycle. In addition, impaired OXPHOS leads to the accumulation of metabolic intermediates, such as lactate, which interferes with normal metabolic processes (Sergio et al., 2021; Abu Shelbayeh et al., 2023).

### 3.2 Mitochondrial biogenesis

Mitochondrial biogenesis refers to the generation and proliferation of new mitochondria within cells, a process essential for maintaining cellular energy supply and metabolic equilibrium. This process includes the replication of mitochondrial DNA, the formation of mitochondrial membranes, and the synthesis and assembly of mitochondrial proteins (Chen L. et al., 2023; Lapatto et al., 2023; Malik et al., 2023). Overconsumption of nutrients results in elevated free fatty acid levels enhanced mitochondrial ROS generation, compromised mitochondrial performance in fat cells, decreased mitochondrial formation and DNA quantity, and lowered β-oxidation rates. Subsequently, fat cell metabolic processes undergo changes, as evidenced by modifications in fat synthesis, breakdown, fatty acid bonding, and the production of lipocalin by adipocytes. These metabolic shifts lead to reduced insulin responsiveness (Bénédicte A. et al., 2021). Obesity leads to downregulation of the gene expression of mitochondrial respiratory complex components, with the protein peroxisome proliferator-activated receptor γ coactivator 1α (PGC-1α) being a key regulator of mitochondrial biogenesis. PGC-1α stimulates mitochondrial creation by interacting with various nuclear receptors and gene transcription regulators, including peroxisome proliferator-activated receptor γ (PPARγ). However, the expression and function of PGC-1α are significantly impaired in individuals with obesity (Rius-Perez et al., 2020).

### 3.3 Mitochondrial dynamics

Mitochondrial dynamics are governed by a series of GTPase proteins. Dynamin-related protein 1 (Drp1) is primarily responsible for the division of mitochondrial membranes, while mitochondrial fission protein 1 (Fis1) also plays a crucial role in regulating mitochondrial fragmentation (Duan et al., 2021; Yu et al., 2021; Huan et al., 2023). On the other hand, GTPase optic atrophy 1 (Opa1) is responsible for the fusion of the inner mitochondrial membrane, and mitochondrial fusion proteins 1 (Mfn1) and 2 (Mfn2) control the fusion of the outer mitochondrial membrane (von der Malsburg et al., 2023). In the obese state, mitochondrial division tends to be overactive, leading to mitochondrial fragmentation and dysfunction. Excessive mitochondrial division not only decreases the number of mitochondria but also increases ROS production, triggering a cellular stress response and metabolic dysregulation (Al Ojaimi et al., 2022). Moreover, reduced mitochondrial fusion limits mitochondrial repair and functional optimization, exacerbating obesity-related metabolic issues (Garcia et al., 2022). Changes in the proteins that regulate mitochondrial structure and function are closely linked to obesity-related mitochondrial dysfunction. Specifically, several studies have shown that decreased expression of OPA1 and Mfn2 is linked to impaired mitochondrial functionality in obesity (Mancini et al., 2019; Zheng et al., 2023). Pereira et al. indicated that deletion of the mitochondrial protease OMA1 disrupts OPA1 processing, affecting metabolic balance and leading to increased fat mass and reduced energy expenditure (Pereira et al., 2021). Similarly, Da et al. reported that Mfn2 deficiency can lead to elevated hydrogen peroxide and ROS levels, contributing to mitochondrial dysfunction in liver and muscle tissues (Da Dalt et al., 2024). Further investigations in HFD-fed mice revealed that changes in mitochondrial dynamics—from fusion to increased fission—are associated with respiratory challenges and reduced ATP production in skeletal muscle, strengthening the connection between impaired mitochondrial dynamics and metabolic disturbances in obesity (Yang H.-M. et al., 2023; Kim et al., 2024).

### 3.4 Mitochondrial autophagy

Mitochondrial autophagy, a crucial process for maintaining mitochondrial quality and function, selectively targets and removes damaged mitochondria. Research has shown that impaired regulation of mitochondrial autophagy in the skeletal muscle of obese individuals leads to a decrease in both the quantity and functionality of mitochondria (Pileggi et al., 2021). Interestingly, while a short-term high-fat diet can trigger mitochondrial autophagy in skeletal muscle, it does not affect mitochondrial respiratory capacity. This phenomenon might be associated with lipid-induced oxidative stress, indicating that mitochondrial autophagy might serve a protective role during the early stages of obesity (Ehrlicher et al., 2021). Additionally, impaired mitochondrial autophagy in adipose tissue may exacerbate the progression of insulin resistance and obesity by influencing the release of adipokines and cytokines (Turchi et al., 2020). Enhanced lipid autophagy and mitochondrial autophagy in brown and beige adipocytes, especially in cold environments, promote adaptive thermogenesis, helping the body cope with cold stimuli (Sakers et al., 2022). Recent studies have also shown that triiodothyronine (T3) modulates mitochondrial homeostasis by inducing lipophagy and mitochondrial autophagy in the liver and skeletal muscle and stimulates thermogenesis (Yau et al., 2021; Hatziagelaki et al., 2022). This mechanism triggers the expression of mitochondrial uncoupling protein 1 (UCP1), enhances autophagy-dependent fatty acid oxidation, and regulates mitochondrial autophagy, functionality, and renewal in brown adipose tissue (BAT) and aging skeletal muscle (Wang et al., 2023). Furthermore, as shown in Table 1, mitochondrial dysfunction presents differently at various stages of obesity.

**TABLE 1:** Mitochondrial dysfunction in different stages of obesity.

Stage of obesity	Main characteristics	Specific manifestations of mitochondrial dysfunction	References
Early Stage	Weight gain, adipocyte hypertrophy	Decreased mitochondrial biogenesis, and impaired oxidative phosphorylation	Baldini et al. (2021)
Intermediate Stage	Chronic low-grade inflammation, onset of insulin resistance	Elevated ROS levels, and dysregulated lipid oxidation, exacerbated insulin resistance	Wang et al. (2021)
Advanced Stage	High risk of metabolic complications	Mitochondrial DNA damage, defective mitophagy, increased apoptosis and necrosis	Montgomery (2019)
Metabolic Disease Stage	Metabolic syndrome, type 2 diabetes, cardiovascular diseases	Tissue-specific mitochondrial dysfunction, chronic inflammation, fibrosis, disrupted metabolic homeostasis	Arruda et al. (2014), Cavaliere et al. (2023)

To address these challenges, antioxidants have been investigated for their potential to reduce oxidative stress and protect mitochondrial function. Among them is mitoquidone (MitoQ), a chemically modified form of coenzyme Q10 that selectively enters mitochondria and accumulates in their inner membrane, thereby safeguarding them from oxidative stress (Chen et al., 2022). Additionally, MitoQ has been shown to normalize metabolic profiles, reduce lipid peroxidation, and restore UCP-2 protein levels to normal values in obese rats (Xu et al., 2021). Research also indicate that MitoQ improves NF-κB activation and mitigates endoplasmic reticulum stress by regulating mitochondrial function (Cojocaru et al., 2023). Water-soluble Szeto-Schiller (SS) peptides, another group of mitochondria-targeted antioxidants, are small molecules that are quickly and selectively taken up into the inner mitochondrial membrane (IMM) across various cell types (Zong et al., 2024). Studies have demonstrated that SS-31 reduces mitochondrial ROS production, suppresses changes in mitochondrial membrane permeability, and safeguards cells from oxidative stress-induced death (Peng et al., 2021; Yang et al., 2021).

## 4 Gut microbiota and its role in obesity

### 4.1 Gut microbiota and its metabolites

The gut microbiota consists of many microorganisms, including archaea, protozoa, fungi, viruses, and bacteria, with bacterial species being the most abundant and extensively studied group (Zhang et al., 2022). The major intestinal bacterial phyla are *Firmicutes, Bacteroidetes, Actinobacteria, Proteobacteria, Fusobacteria*, and *Verrucromicrobia*, with *Firmicutes* and *Bacteroidetes* being 90% of the total bacterial population. Notably, although certain bacteria are less abundant, this does not necessarily indicate a secondary functional role (Fassarella et al., 2021). Different microbial concentration gradients are observed throughout the gut, and understanding this distribution is crucial for investigating the impact of the gut microbiota on the host’s health. The lower microbial density in the upper gastrointestinal tract helps prevent pathogen colonization. In contrast, the more abundant microbiota in the lower intestine facilitates the breakdown of complex carbohydrates and the production of SCFAs, which are metabolic byproducts essential for the host’s energy metabolism and intestinal health (Wen et al., 2022).

The intestinal microbial community begins to form at birth and potentially even earlier in the womb. It is influenced by numerous factors, such as the delivery method, infant nutrition, lifestyle choices, genetic predisposition, medication use, and dietary habits (Valles-Colomer et al., 2023). Typically, the intestinal microbiota attains an adult-like configuration within the first 5 years of life, though it continues to evolve dynamically throughout an individual’s lifetime (Roswall et al., 2021). The symbiotic relationships between humans and their gut microbiota are essential for maintaining health. The intestinal microbiota plays a vital role in various physiological processes, including preserving the integrity of the intestinal barrier, providing against pathogens, and modulating immune responses and diverse metabolic functions (Lee et al., 2022; Loh et al., 2024; Zhu et al., 2024).

Along with the importance of the gut microbiota, the byproducts of microbial metabolism also play crucial roles. The host absorbs and utilizes the gut microbiota metabolites SCFAs, providing additional energy and regulating host metabolic processes through various mechanisms (Xiong et al., 2022). Previous studies have extensively documented key bacteria involved in SCFA production’s metagenomic characteristics and associated pathways (Tsukuda et al., 2021; Frolova et al., 2022).

Currently, *Akkermansia muciniphila*, *Bacteroides* spp., *Bacteroides vulgatus*, *Bifidobacterium* spp., *Lactobacillus* spp., and *Prevotella* spp. are the primary producers of acetate and propionate, while *Coprococcus* spp., *Eubacterium* spp., *Faecalibacterium prausnitzii, and Roseburia* spp. are the main butyrate-producing bacteria (Kim et al., 2020; Lordan et al., 2020). In addition to SCFAs, the gut microbiota can influence host metabolic processes through metabolites such as SBAs, tryptophan metabolites, and hydrogen sulfide (H_2_S). By metabolizing primary bile acids and producing tryptophan metabolites via the tryptophan metabolic pathway, *Bacteroides* and *Clostridium* are converted to SBAs (Matthew F. et al., 2020; Xiaomin S. et al., 2022; Xiaomin T. et al., 2022). *Desulfovibrio* and *Bilophila* species produce H_2_S through cysteine degradation, while *Fusobacterium nucleatum* generates H_2_S during digestion (DJ et al., 2021; Matthew W. et al., 2023).

### 4.2 Gut microbiota and its metabolites in obesity

HFD consumption, lifestyle choices, antibiotics use, and psychological or physical stress are key factors that alter the composition of intestinal microbial and its metabolic products, potentially disrupting gut homeostasis, leading to dysbiosis (IJ et al., 2021; Francois et al., 2022). Extensive research using both animal models and human participants has demonstrated that imbalances in gut microbiota interfere with energy metabolism and lead to obesity (Lee et al., 2020; Wachsmuth et al., 2022; Takeuchi et al., 2023). Certain bacterial species have been identified as either harmful or beneficial in the progression of these conditions. Compared to individuals with normal weight, obese individuals exhibit reduced microbial diversity and richness in their intestines, along with structural changes in the microbiome and a disproportionate *Firmicutes-*to-*Bacteroidetes* ratio, typically characterized by an increase in *Firmicutes* and a decrease in *Bacteroidetes* (Manor et al., 2020a). Similar findings have been observed in animal studies, where the expression of *Lactobacillus* and *Bifidobacterium*, among others, was reduced in the gut of obese mice (Huo et al., 2020; Zhihao L. et al., 2022). Conversely, in cases of obesity induced by excessive fat consumption, elevated levels of bacterial lipopolysaccharide (LPS) have been reported (Ramos-Romero et al., 2020). This condition disrupts intestinal structure, increases intestinal permeability, and allows significant amounts of LPS to enter the bloodstream, triggering chronic inflammatory and accelerating the progression of obesity (Camilleri, 2023; Di Vincenzo et al., 2024).

Given the gut microbiota’s central role in obesity and its associated metabolic dysregulation, targeted modulation of the intestinal ecosystem emerges as a promising therapeutic avenue. The microbiota’s significant adaptability in its makeup and function makes it a promising target for treating and preventing various health conditions. Strategies such as probiotics, prebiotics, or symbiotics are commonly explored. In the field of functional foods and nutritional supplements, *Bifidobacterium* and *Lactobacillus* are among the most frequently used probiotics (Sharma et al., 2021). Notably, the South Korean Food and Drug Administration has approved *Lactobacillus gasseri* BNR17 as a functional component for reducing adipose tissue (Kim et al., 2018). New-generation probiotics, such as *F. prausnitzii* strains, are also gaining attention, as they are commonly found in the microbiota of healthy individuals. Studies have linked a decline in these strains to a higher likelihood of an increased risk of developing obesity (Kallassy et al., 2023; Yang M. et al., 2023). *Faecalibacterium prausnitzii* exhibits anti-inflammatory properties, as its supernatant suppresses the NF-κB pathway *in vitro* and *in vivo* (Auger et al., 2022). It also synthesizes butyrate and other SCFAs, contributing to its beneficial effects (NM et al., 2017). Prebiotics, defined as nutritional components that undergo selective fermentation, can potentially modify the intestinal microbiota’s composition or functionality, leading to beneficial outcomes for the host’s health (Miyamoto et al., 2023). Most evidence supporting their efficacy stems from studies on dietary constituents, primarily categorized into two chemical groups: inulin-type fructans and galacto-oligosaccharides (GOSs) (Piotr P. et al., 2022). Fibers rich in prebiotics stimulate enteroendocrine L-cell development and elevate the anorexigenic peptides PYY and GLP1 concentrations in both the intestinal lumen and bloodstream, consequently diminishing caloric consumption (Akhlaghi, 2024). Human-based research has demonstrated that consuming diets abundant in prebiotics correlates with decreased food consumption, lowered adipose tissue, and minimized weight increase, particularly among individuals with obesity (Huwiler et al., 2022; Jagielski et al., 2023). Synbiotics, which combine prebiotics and probiotics, offer a promising strategy for addressing imbalances in the intestinal microbiota (Saadati et al., 2024). Dietary symbiotics, incorporating carefully selected bacterial strains—such as *L. gasseri* variants known for their weight-reducing and anti-inflammatory properties, paired with galactomannan or inulin fibers may provide enhanced benefits for tackling obesity by promoting SCFA production and restructuring the microbiome (Li X. et al., 2023). Studies have shown a significant decrease in obesity among mice on high-fat diets when administered a symbiotic blend of D-allulose and the probiotic strains *Lactobacillus sakei* and *Leuconostoc kimchii* (Choi et al., 2018). Moreover, introducing symbiotics to mice early in life modulates their gut microbiota, protecting against diet-related obesity as they age (Kang et al., 2023).

The precise function of gut microbiota in the onset of metabolic disorders remains unclear. Recent research has emphasized the connections between microbiota diversity, composition, and mitochondrial function. Importantly, gut microbiota and their metabolic byproducts significantly regulate different factors, transcriptional coactivators, and enzymatic processes that affect mitochondrial functionality (Qi et al., 2022). In addition, abnormal mitochondrial function may cause excessive ROS release, dysregulation of glucolipid metabolism, and abnormal adipose tissue function, which affect gut microbiota homeostasis and result in bodily imbalance, triggering obesity (Zhu et al., 2023). The following sections will delve into the complex interplay between gut microbiota and mitochondrial operations, exploring potential pathways through which these interactions could be leveraged to develop innovative approaches for obesity management.

## 5 Gut microbiota–mitochondrial crosstalk in obesity

### 5.1 Role of mitochondrial energy metabolism in the gut microbiota

Emerging research highlights an interactive relationship between mitochondrial energy metabolism and the gut microbiota. Efficient mitochondrial energy production relies on the integration of fatty acid oxidation, the tricarboxylic acid cycle (TCA cycle), and OXPHOS. OXPHOS utilizes NADH and FADH2, produced via fatty acid oxidation and the TCA cycle, to generate significant amounts of ATP (Guo et al., 2023). Key byproducts of this process are essential in regulating the gut environment and shaping the gut microbiota, thus influencing obesity development (Wan et al., 2020; Bhattacharjee et al., 2023). ROS are produced during mitochondrial OXPHOS. Obese individuals exhibit markedly altered proportions of *Bacteroides* and *Firmicutes* in their gut microbiota, which is closely associated with disrupted mitochondrial OXPHOS and ROS production (Jinhua et al., 2022). Interestingly, ROS serves a dual role in intestinal inflammation, functioning as important signaling molecules produced by mitochondria during OXPHOS. Moderate levels of ROS support intestinal homeostasis by regulating cell proliferation, differentiation, and immune responses. Studies have also demonstrated that ROS can activate the Nrf2 signaling pathway, promoting the expression of antioxidant genes and enhancing cellular antioxidant defenses (He et al., 2022).

However, in obese individuals, excessive ROS production leads to oxidative stress, causing damage to proteins, lipids, and DNA (Abdolmaleky and Zhou, 2024). This disrupts intestinal barrier integrity, triggers an inflammatory response, and alters the gut microenvironment, allowing the colonization of harmful bacteria, such as *Enterobacteriaceae*, while inhibiting the growth of beneficial bacteria like *Bifidobacteria* (Jeong and Kang, 2023). Organic acids, natural byproducts of the TCA cycle, inhibit the growth of *Escherichia coli* and *Salmonella* by lowering intestinal pH, while promoting the proliferation of *Lactobacillus* and *Bifidobacterium*. A HFD raises intestinal pH, disrupting gut microbiota equilibrium and affecting the gut’s metabolic state (Guo et al., 2021; Ekkachai et al., 2023). Ketone bodies, including β-hydroxybutyrate (BHB) and acetoacetate, are produced by mitochondria during fatty acid oxidation. These ketones are not only crucial energy metabolites but also important signaling molecules. Metabolism and inflammatory responses in host cells are regulated by BHB through activation of the G protein-coupled receptor GPR109A and suppression of NF-κB and NLRP3 inflammasomes, leading to reduced intestinal inflammation and improved balance of the gut microbiota (Valentina et al., 2022). In an *in vitro* human colon microbiota model, BHB supplementation increased butyric acid production (Kengo et al., 2020). Furthermore, BHB influences cellular gene expression and metabolic pathways by inhibiting histone deacetylase (HDACs) and modifying SCFA production, which subsequently impacts the host’s energy balance and insulin sensitivity. This process is essential for maintaining the diversity and functionality of the gut microbiota, particularly in metabolic disorders related to obesity (KB et al., 2021).

Moreover, mitochondrial dysfunction exacerbates obesity-related metabolic dysregulation by influencing the gut microbiota through immune and neural pathways. Regarding immune regulation, mitochondrial dysfunction causes the release of mitochondrial DNA into the cytoplasm, where it acts as a pathogen-associated molecular pattern. This mtDNA then interacts with Toll-like receptor 9 (TLR9), triggering a MyD88-dependent signaling cascade that stimulates the production of inflammatory factors, altering the intestinal microenvironment and impacting the composition of the intestinal microbiota (Ren et al., 2020). Additional research has shown that depletion of the mitochondrial membrane potential leads to intracellular potassium ion outflow, subsequently activating various inflammatory vesicles, such as NLRP3. Potassium ion efflux not only activates these inflammatory vesicles but also compromises the integrity of intestinal epithelial cells, impairing gut barrier function and increasing permeability, which further disrupts microbiota homeostasis (Diwakar et al., 2021; Wei et al., 2021; Matthew Y. et al., 2023). Moreover, the state of mitochondrial metabolism significantly affects dendritic cell function. Mitochondrial stress signals can modulate the antigen-presenting function and cytokine secretion of dendritic cells, thereby affecting the immune response in the gut (Adamik et al., 2022). In obesity, mitochondrial stress promotes increased secretion of proinflammatory cytokines by dendritic cells, resulting in an increase in Th17 cells, which are crucial in intestinal inflammation (van der Zande et al., 2023). In terms of neuromodulation, mitochondrial dysfunction disrupts the synthesis and degradation of serotonin and dopamine, key neurotransmitters that regulate intestinal function, affecting intestinal peristalsis and secretion. This in turn alters the composition of the microbiota (Xie et al., 2020). Imbalances in the autonomic nervous system, particularly an underactive or overactive sympathetic and parasympathetic response, also affect the gut microbiota in the obese state. The vagal nerve regulates gut peristalsis, secretion, and barrier function by releasing acetylcholine. In obese individuals, decreased vagal activity leads to increased colonization of *E. coli* and *Salmonella*, while inhibiting the growth of *Lactobacillus* and *Bifidobacterium* (Bénédicte et al., 2017). Additionally, the enteric nervous system (ENS), often referred to as the “second brain,” directly controls intestinal behavior. Mitochondrial dysfunction in obese individuals impairs the energy-dependent functions of ENS neurons leading to changes in intestinal movement and enzymatic secretion. A decrease in digestive enzyme secretion can lead to incomplete food digestion, increased retention of undigested material in the intestine, and provide nutrients for harmful bacteria. Conversely, excessive secretion may dilute intestinal contents, affecting the growth of beneficial bacteria (Bénédicte N. et al., 2021). In obese individuals, ENS dysfunction also compromises epithelial cell function and reduces tight junction protein expression, enhancing intestinal permeability. This impaired barrier function enables the increased entry of pathogenic bacteria and toxins into the bloodstream, triggering a widespread inflammatory response and further destabilizing the gut microbiota equilibrium (Bénédicte et al., 2020).

In summary, mitochondria regulate the gut microbiota through their metabolic products while maintaining gut microbiota balance by preventing oxidative stress, immune dysregulation, and neuroregulatory imbalances. This underscores the impact of mitochondrial dysfunction on gut microbiota homeostasis and its close association with the development of obesity.

### 5.2 The role of gut microbiota in mitochondrial energy metabolism

**FIGURE 2:**
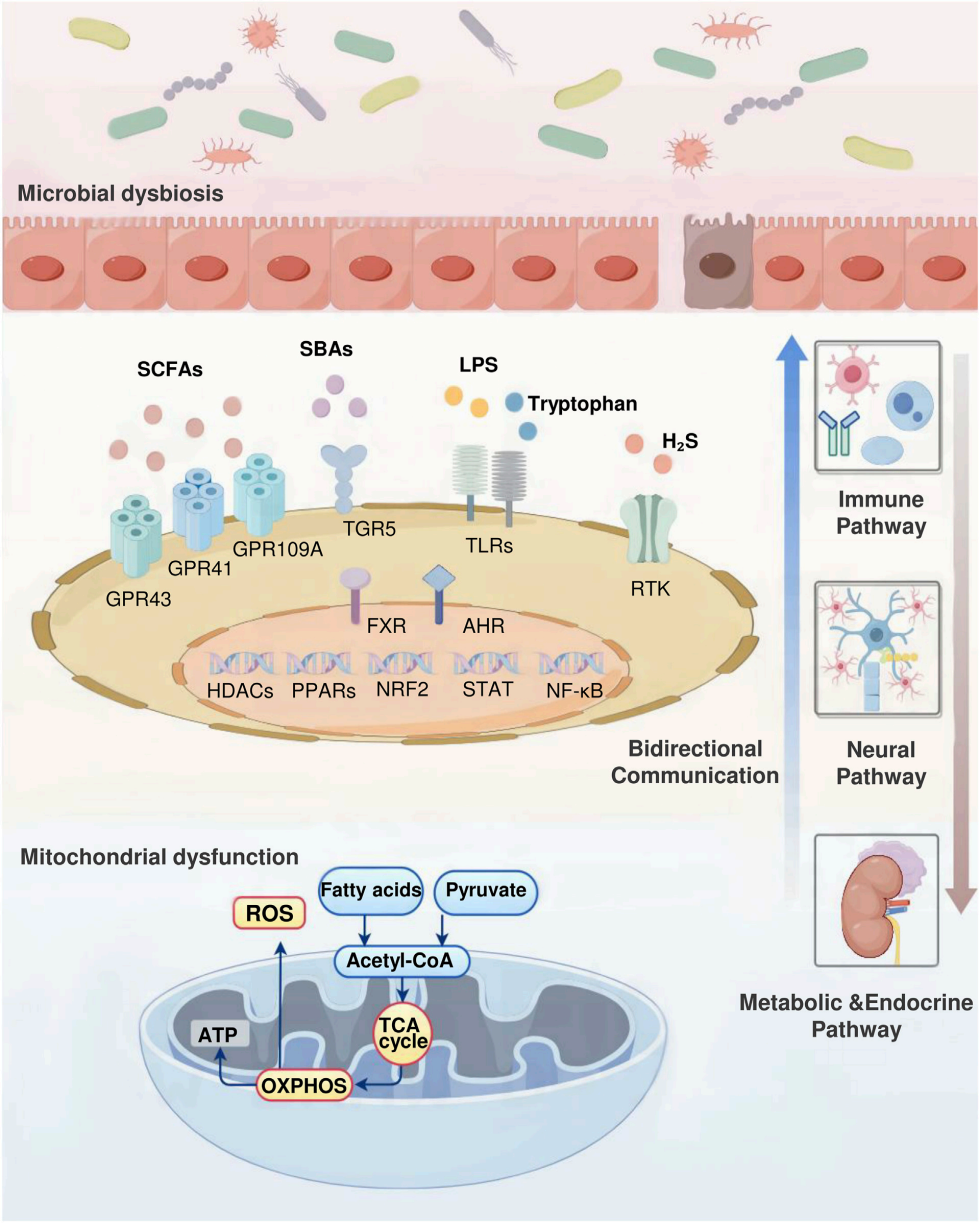
Gut microbiota–mitochondrial crosstalk in obesity. The complex interactions between the gut microbiota and mitochondrial function underscore their key role in regulating obesity. This bidirectional communication occurs through immune, neural, and metabolic-endocrine pathways. Gut microbial metabolites such as SCFAs, SBAs, tryptophan, H2S, and LPS maintain body homeostasis and directly influence the development of obesity by regulating mitochondrial activity. Conversely, mitochondrial energy metabolites such as ROS, organic acids, and ketone bodies alter the intestinal microenvironment, affecting microbial composition. This process involves multiple signaling molecules. Receptors including GPR43, GPR41, GPR109A, FXR, TGR5, TLRs, AHR, RTK. Key transcription factors include Nrf2, NF-κB, STAT, and PPARs. Enzymes including HDACs.

Changes in gut microbiota composition can lead to alterations in environmental metabolites, shedding light on the mechanisms behind obesity development. Extensive studies using germ-free mice that were inoculated with gut microbiota from obese individuals have strongly confirmed the influence of gut microbiota on the host’s energy metabolism, glucose tolerance, and insulin resistance (Diwakar et al., 2022). Additionally, metabolites produced by gut microbiota play a vital role in maintaining homeostasis in the body and affect the progression of obesity through their impact on mitochondrial function. The following section explores the key metabolites derived from gut microbiota that have recently been associated with the advancement of obesity (Figure 2).

#### 5.2.1 SCFAs

Among the metabolites produced by gut microbiota, SCFAs primarily include acetic, propionic, and butyric acids. Acetic acid regulates adipocytes’ mitochondrial function and fatty acid oxidation by activating the GPR43 receptor. This process increases cAMP production, which, in turn, activates protein kinase A (PKA), influencing fat accumulation and energy metabolism (Manor et al., 2020b). Acetic acid modulates glucose metabolism in obesity-related cell types, including adipocytes and macrophages, via the GPR43 receptor (Li et al., 2024). Propionic acid’s activation of GPR41 and GPR43 receptors triggers the ERK and AMPK signaling cascades, stimulating mitochondrial biogenesis and lipolysis. This process enhances insulin sensitivity, mitigates oxidative stress, and reduces mitochondrial damage, thereby combating the metabolic disorders associated with obesity (Hiroki et al., 2019; Xiaomin W. et al., 2022). Studies have revealed that propionate metabolism involves acetyl coenzyme A synthase short-chain family member 3 (ACSS3) in the IMM. ACSS3 plays a crucial role in propionate metabolism, and ACSS3 deficiencies lead to serum propionic acid accumulation, causing reduced BAT, increased white adipose tissue (WAT), and insulin resistance (Zhihao J. et al., 2022). Research has also shown that propionic acid stimulates thermogenic responses and promotes the browning of adipose tissue through various mechanisms, including the upregulation of PGC-1α expression, a key regulator of mitochondrial biosynthesis (CC et al., 2021). Among the SCFAs, butyric acid improves mitochondrial function through GPR109A receptor activation, AMPK signaling pathway modulation, enhanced mitochondrial autophagy, and attenuation of inflammatory responses (Xaomin et al., 2020). Additionally, research has shown that butyrate enhances UCP1-induced BAT thermogenesis and WAT browning by upregulating PGC-1α expression (YM et al., 2020).

#### 5.2.2 SBAs

In addition to SCFAs, other microbial-derived metabolites are associated with metabolic processes and obesity. SBAs, including deoxycholic acid (DCA) and ursodeoxycholic acid (UDCA), influence mitochondrial energy metabolism primarily through the actions of the farnesoid X receptor (FXR) and the G protein-coupled bile acid receptor 5 (TGR5). FXR reduces hepatic lipid accumulation by suppressing SREBP-1c, regulates hepatic gluconeogenesis by enhancing FGF15/19 and PPARα expression, lowers blood glucose levels, and stimulates mitochondrial biogenesis and fatty acid oxidation. These actions collectively reduce lipid accumulation and enhance insulin sensitivity (JYL et al., 2020; Lulu et al., 2021; Matthew et al., 2021). TGR5, through its regulation of the AMPK signaling pathway, promotes BAT thermogenesis, increases energy expenditure, and enhances mitochondrial OXPHOS (SR et al., 2020).

#### 5.2.3 Tryptophan and H_2_S

Tryptophan metabolites primarily include indole propionic acid (IPA), indole-3-acetic acid (IAA), kynurenine, and quinolinic acid (QA). IPA promotes fat metabolism and reduces fat accumulation by stimulating the PPARγ-AMPK pathway, which enhances mitochondrial OXPHOS, promotes lipid metabolism, and decreases fat accumulation (Piotr K. et al., 2022). IAA regulates intestinal immune responses, reduces inflammation-related metabolic disorders, and influences mitochondrial biogenesis and autophagy by activating the TLR4-JNK pathway (Ruiz et al., 2020; Chowdhury et al., 2021). The kynurenine pathway produces metabolites that activate the aromatic hydrocarbon receptor, regulate gene expression, influence immune and inflammatory responses, and promote mitochondrial biogenesis (Ren et al., 2022). QA modulates cellular proliferation and metabolic processes by stimulating the mammalian target of rapamycin protein (mTOR) signaling cascade, improving mitochondrial performance, and regulating lipid biosynthesis and energy utilization (Bénédicte H. et al., 2021).

Additionally, hydrogen sulfide (H_2_S), a metabolite of the intestinal microbiota, affects mitochondrial function. H_2_S is produced by intestinal anaerobic sulfate-reducing bacteria, and high-fat diets lead to an increase in these bacteria in the gut of obese rats, resulting in elevated H_2_S production (Francois et al., 2021). H_2_S comprehensively modulates cellular metabolism, mitochondrial performance, and inflammatory pathways by stimulating the PI3K/Akt and AMPK signaling pathways, while concurrently suppressing the NF-κB pathway (Diwakar et al., 2020; Bénédicte W. et al., 2021; Alex et al., 2022).

#### 5.2.4 Lipopolysaccharides

Under normal physiological conditions, LPS generated from intestinal microbiota metabolism is nonpathogenic. However, prolonged stimulation induces macrophages to secrete inflammatory mediators, triggering ROS generation in the jejunum and ileum. This cascade results in mitochondrial enlargement, reduced membrane potential, structural abnormalities, and impaired functionality of intestinal epithelial cells (Matthew and PY, 2020). Additional research has demonstrated that LPS increases intestinal permeability, allowing more LPS molecules to enter the bloodstream and activate a systemic immune response. In the bloodstream, LPS forms complexes with LPS-binding proteins and CD14, engaging Toll-like receptor 4 (TLR4). This interaction triggers various inflammatory signaling pathways, leading to proinflammatory cytokines like IL-6 and TNF-α secretion (He et al., 2019). These mediators subsequently compromise mitochondrial performance and decrease metabolic energy efficiency (Matthew X. et al., 2020; Zhang X. et al., 2021). Additionally, LPS promotes adipose tissue proliferation and inflammation by activating the JAK/STAT pathway (CC et al., 2019).

## 6 Therapeutic potential of targeting the gut microbiota and mitochondria from traditional Chinese medicine

**FIGURE 3:**
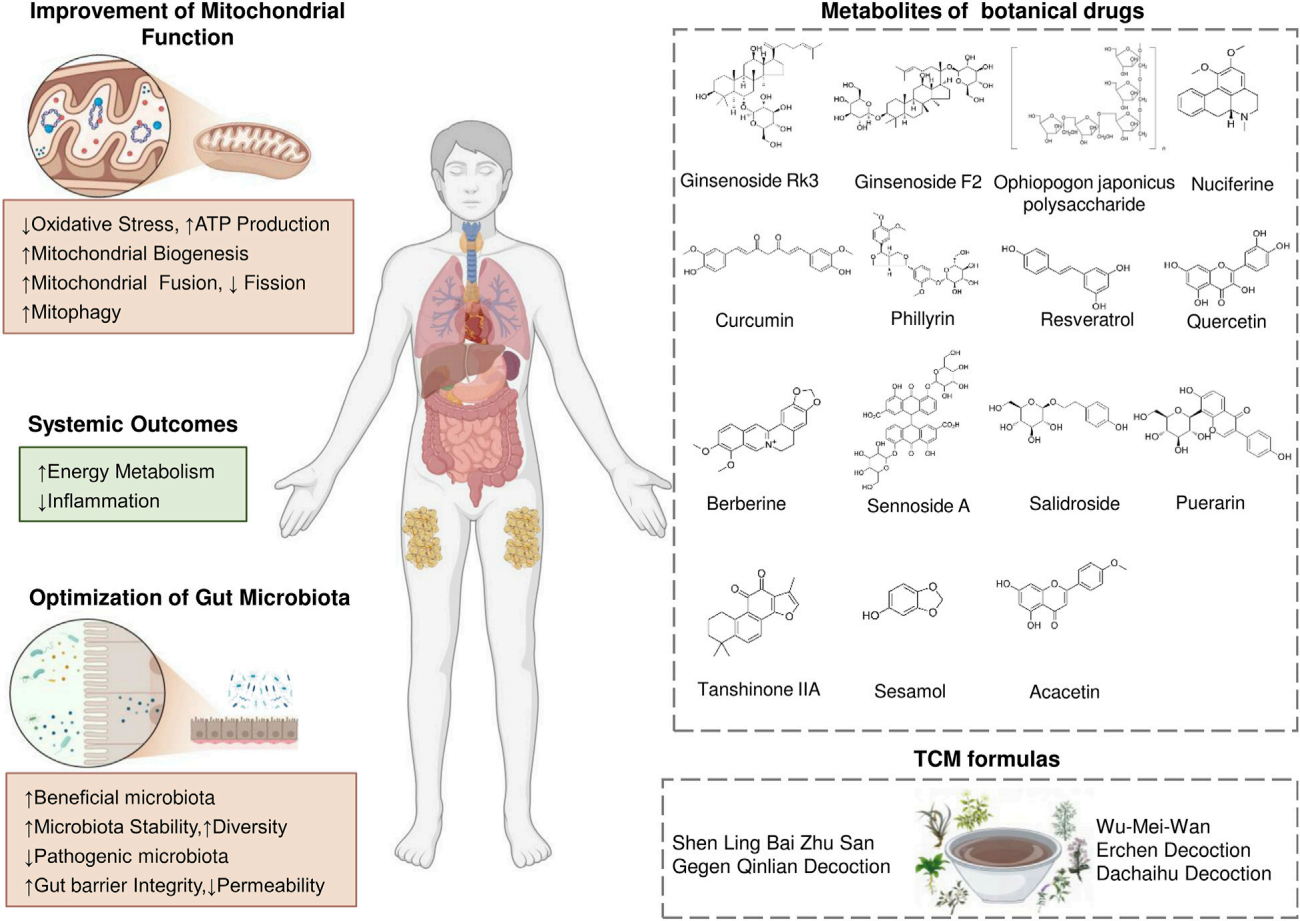
Therapeutic Strategies Targeting the Gut Microbiota–Mitochondrial Crosstalk for Obesity. Traditional Chinese medicine works synergistically to enhance mitochondria-gut crosstalk. This mutual reinforcement between improved mitochondrial function and a healthier gut microbiota leads to better energy metabolism, enhanced insulin sensitivity, and effective inflammation control, offering a comprehensive strategy for obesity management.

Accumulating evidence suggests that therapies targeting the interplay between mitochondria and the microbiota may offer a new approach to treating these diseases or reducing their complications, providing a broader range of effects than targeting mitochondria or the microbiota alone. Recent studies have uncovered multiple molecular mechanisms mediating the gut microbiota and mitochondria interaction, potentially paving the way for the development of precisely targeted therapeutic interventions. Conversely, alterations in mitochondrial activity can influence the microbial community, thereby affecting disease progression and highlighting the gut microbiota and mitochondria bidirectional communication. In this context, TCM formulas and metabolites of botanical drugs which are guided by TCM theory, offer unique advantages (Law et al., 2022; Zhang Q. et al., 2023). These therapeutic providing integrative strategies that align with the complexity of this bidirectional interaction (Figure 3).

Contemporary research has increasingly shown that metabolites of botanical drugs can contribute to obesity prevention and management by influencing mitochondrial energy processes and the gut microbiota (Cheng et al., 2023; Luo et al., 2023; Zhi et al., 2024). The following metabolites of botanical drugs that have demonstrated significant effects in relevant studies are shown in Table 2.

**TABLE 2:** Metabolites of  botanical drugs for the treatment of obesity.

Sources information	Plant metabolites	Types of study	Experiment object	Dosage	Intervention mode	Modeling methods	Effects on Gut Microbiota–Mitochondrial Crosstalk	Mechanisms	Metabolic Outcomes	References
Panax ginseng C.A. Mey. [Araliaceae; Ginseng Radix et Rhizoma]	Ginsenoside Rk3	*In vivo*	Male C57BL/6JFandd mice	20,60 mg/kg	Gavage 2 weeks	Antibiotic-induced gut microbiota dysbiosis model (3 g/kg lincomycin administration)	↑ *Bacteroides, Alloprevotella, and Blautia*,↓ *Firmicutes*; ↑Mitochondrial membrane integrity	↑ AMPK/Akt; ↓ TNF-α, IL-1β, IL-6, IL-17; ↓NLRP3	↓Inflammatory response,↑SCFAs production,↓Lipid accumulation	Bai et al. (2021)
Panax ginseng C.A. Mey. [Araliaceae; Ginseng Radix et Rhizoma]	Ginsenoside F2	*In vivo* and *In vitro*	*In vivo*:Male C57BL/6J mice *In vitro:*3T3-L1 Cells	*In vivo:*50,100 mg/kg; *In vitro:*12.5, 25, 50, 100 µM	Gavage 4 weeks	*In vivo:*High-fat diet *In vitro*:3T3-L1 preadipocyte differentiation model	↑Mitochondrial biogenesis,↑ Mitochondrial function	↑ AMPK/ACC phosphorylation; ↓ PPARγ and C/EBPα	↓Adipocyte differentiation,↑Energy metabolism,↓ oxidative stress	Zhou et al. (2021)
Ophiopogon japonicus (Thunb.) Ker Gawl. [Asparagaceae; Ophiopogonis radix]	*Ophiopogon japonicus* polysaccharide	*In vivo*	Male C57BL/6J mice	300 mg/kg	Gavage, 12 weeks	High-fat diet	↑ *Lactobacillus*;↑Activity of mitochondrial respiratory chain complexes	↑ AMPK and Nrf2; ↓NF-κB	↑Oxygen consumption,↑Energy expenditure,↓Lipid metabolism disorder	Shi et al. (2015)
Curcuma longa L. [Zingiberaceae; Curcumae longa rhizoma]	Curcumin	*In vivo* and *In* *vitro*	*In vivo:*Male C57BL/6J mice *In vitro:*3T3-L1 Cells	*In vivo*:50 mg/kg;*In vitro*:10–35 µM	Gavage 8 weeks	*In vivo:*High-fat diet *In vitro:*3T3-L1 preadipocyte differentiation model	↑ Mitochondrial oxygen consumption,↑ Mitochondrial respiration and ATP production	PPARγ Pathway Activation; ↑ Browning of WAT; ↑ UCP1, PGC-1α, PRDM16	↓ Body weight, ↓ Fat accumulation, ↓ Inflammation, ↑ Energy expenditure, ↑ Insulin sensitivity	Zhao et al. (2021a)
		*In vitro*	Male C57BL/6J mice	0.2% (w/w) in diet	Oral supplementation 10 weeks	High-fat diet	↓ *Firmicutes/Bacteroidetes* ratio, ↓ *Desulfovibrio*,↑ *Bacteroides*, *Parabacteroides*, *Alistipes* and *Alloprevotella*	↑SCFA; ↓LPS	↓ Body weight, ↓ Hepatic steatosis, ↓ Inflammation, ↑ Insulin sensitivity	Li et al. (2021b)
Forsythia suspensa (Thunb.) Vahl [Oleaceae; Forsythiae fructus]	Phillyrin	*In vivo*	Male C57BL/6J mice	25,50 mg/kg	Gavage 8 weeks	High-fat diet	↓Mitochondrial membrane permeability; ↑Fatty acid oxidation	↑ AMPK	↓Lipid accumulation	Fang et al. (2022)
Nelumbo nucifera Gaertn. [Nelumbonaceae; Nelumbinis folium]	Nuciferine	*In vivo*	Male Sprague-Dawley rats	10 mg/kg	Gavage 8 weeks	High-fat diet	↓ *Firmicutes/Bacteroidetes* ratio, ↓ *Desulfovibrio*	↓SREBP-1, PPARγ, FAS; ↑PPARα	↓Obesity,Fat Accumulation; ↑ Insulin sensitivity	Wang et al. (2020b)
Reynoutria japonica Houtt. [Polygonaceae; Polygoni cuspidati rhizoma et radix]	Resveratrol	*In vivo*	Male C57BL/6J mice	300 mg/kg	Gavage 16 weeks	High-fat diet	↑ *Blautia,* ↓ *Desulfovibrio*, ↓ *Lachnospiraceae_NK4A136_group;*↓ Oxidative stress	Influenced metabolic pathways	↓ Body weight, ↓ Fat accumulation, ↓ Inflammation, ↑ Insulin sensitivity	Wang et al. (2020a)
Scutellaria baicalensis Georgi [Lamiaceae; Scutellariae radix]	Quercetin	*In vivo*	Spotted seabass (Lateolabrax maculatus)	0.5 g/kg、1.0 g/kg	Oral supplementation 8 weeks	High-fat diet	↑ Mitochondrial biogenesis, ↑ Mitophagy, ↓ Endoplasmic reticulum stress	↑ PGC-1α,PINK1; ↓ ATF6,IRE1	↓ Liver triglycerides, ↓ Fat accumulation,↑ Antioxidant capacity	Dong et al. (2021)
		*In vivo*	Male C57BL/6J mice	50 mg/kg	Gavage 20 weeks	High-fat diet	↑ A*kkermansia, Coprococcus_1, Lactococcus* and *Allobaculum*,↓ *Adlercreutzia*	↓ TLR4-MyD88-NF-κB signaling; ↓TNF-α, IL-6, IL-1β	↓ Body weight, ↓ Fat accumulation, ↓ Inflammation, ↓ Insulin resistance	Su et al. (2022)
Coptis chinensis Franch. [Ranunculaceae; Coptidis rhizoma]	Berberine	*In vivo*	Male C57BL/6J mice	100 μg/kg	Gavage 15 weeks	High-fat diet	↑ *Akkermansia muciniphila*, ↑ SCFA-producing bacteria (*Butyricimonas*, *Eubacterium*, *Clostridium*),↓ *Firmicutes/Bacteroidetes* ratio	↓ TLR4-MyD88-NF-κB signaling; ↓ TNF-α, IL-6, iNOS	↓ Insulin resistance,↓ LPS levels,↓ Body weight	Li et al. (2022)
		*In vivo*	Sprague-Dawley rats	100 mg/kg	Oral supplementation 4 weeks	High-fat diet	↑Mitochondrial biogenesis,↓ Oxidative stress,↑ATP,↑ Mitochondrial calcium retention	↑SirT3	↑ Insulin sensitivity,↓ Liver triglycerides, ↓ Hepatic steatosis	Teodoro et al. (2013)
Rheum palmatum L. [Polygonaceae; Rhei radix et rhizoma]	Sennoside A	*In vivo*	Male C57BL/6J mice	30 mg/kg	Oral supplementation 8 weeks	High-fat diet	↑ *Akkermansia muciniphila,*↓ *Firmicutes/Bacteroidetes* ratio; ↑ Mitochondrial function	↑GLP-1; ↑SCFA production	↑ Insulin sensitivity,↑ Energy metabolism	Le et al. (2019)
Rhodiola crenulata (Hook. f. et Thoms.)H. Ohba [Crassulaceae; Rhodiae crenulatae radix et rhizoma]	Salidroside	*In vivo*	Male C57BL/6J mice	15 mg/kg	Gavage 8 weeks	High-fat diet	*↓* *Lachnospiraceae* *, Alistipes finegoldii, Bacteroides sartorii*;*↓* Oxidative stress	↓SREBP-1c, FAS, ACC-1	↓ Insulin resistance,↓Lipid accumulation	Liu et al. (2023)
Pueraria montana var. lobata (Willd.) Maesen and S.M.Almeida ex Sanjappa and Predeep [Fabaceae; Puerariae lobatae radix]	Puerarin	*In vivo*	Male C57BL/6J mice	100 mg/kg	Gavage 4 weeks	High-fat diet	↓*Proteobacteria*, *Bacteroidetes*,↑*Akkermansia muciniphila*, *Clostridium celatum*;↑Mitochondrial function and mitophagy	↓ CYP7A1; ↑FXR, BSEP	↓Metabolic disorders	Yang et al. (2024)
Salvia miltiorrhiza Bunge [Lamiaceae; Salviae miltiorrhizae radix et rhizoma]	Tanshinone IIA	*In vivo*	Male C57BL/6J mice	15 g/kg	Gavage 8 weeks	High-fat diet	*↓ Firmicutes, ↑ Bacteroidota, ↑ Verrucomicrobiota*	Tanshinones activate TFEB nuclear translocation	↑Energy expenditure,↑Insulin sensitivity	Zheng et al. (2024)
		*In vivo* and *In vitro*	*In vivo*:Male C57BL/KsJ-Lepr^−/−^ (db/db) mice, Ucp1^−/−^ mice *In* *vitro*:3T3-L1 Cells	*In vivo*:30 mg/kg;*In vitro*:10, 30, 50 nM	Gavage 8 weeks	High-fat diet	↑ Mitochondrial content in adipocytes,↑ Mitochondrial activity; ↑ UCP1, PGC-1α	AMPK-PGC-1α Pathway Activation		Ma et al. (2022)
Sesamum indicum L. [Pedaliaceae; Sesami semen nigrum]	Sesamol	*In vivo and* *In vitro*	*In vivo*:Male C57BL/6J mice *In vitro*:3T3-L1 Cells	*In vivo*:100 mg/kg *In vitro*:12.5, 25, 50 µM	Gavage 8 weeks	*In vivo:*High-fat diet*;In vitro:*3T3-L1 preadipocyte differentiation model	↑ Mitochondrial biogenesis,↑ Mitochondrial number in adipocytes	β3-AR/PKA Pathway Activation; ↑ UCP1, PGC-1α, NRF1, TFAM; ↓Mitophagy	↓ Body fat; ↓ Serum triglycerides (TG), total cholesterol (TC); ↑ Energy expenditure through increased thermogenesis; ↓Lipid accumulation in adipocytes	Lin et al. (2021)
		*In vivo*	Male C57BL/6J mice	0.2% (w/w) in diet	Gavage 2 weeks	High-fat diet	↑ *Bifidobacterium*, *Akkermansia*, ↓ *Dorea*, *Sutterella*	↑Antioxidant enzyme activities; ↓ MDA; ↑ GSH	↓ Liver lipid accumulation; ↓ Oxidative stress in liver and colon	Wang et al. (2022)
Scutellaria baicalensis Georgi [Lamiaceae; Scutellariae radix]	Acacetin	*In vivo* and *In vitro*	*In vivo*:Male C57BL/6J mice *In vitro*:3T3-L1 Cells	*In vivo*:20 mg/kg *In vitro*:20 μmol/L and 40 μmol/L	*In vivo*:Intraperitoneal injection	*In vivo:*High-fat diet *In vitro:*3T3-L1 preadipocyte differentiation model	↑ Mitochondrial content,↑ Mitochondrial respiratory function; ↓ Lipid accumulation in adipocytes	↑ UCP1, PRDM16, PGC1-α; ↑ cAMP; Activation of AC-cAMP-PKA pathway	↓ Body fat and weight; ↓ Lipid accumulation; Improved glucose and lipid metabolism	Zhang et al. (2023b)

While the therapeutic potential of metabolites of botanical drugs offers a holistic approach to obesity treatment, contemporary research has also highlighted TCM formulas, through their synergistic interactions and multi-target effects (Zhang C. H. et al., 2021). Table 3 summarizes the potential mechanisms of the TCM formulas in detail. Shenling Baizhu San (SLBZS) has the capacity to modulate the proportion of *Bacteroidetes* to *Firmicutes*, enhance the presence of beneficial *Bifidobacterium*, decrease LPS expression, mitigate systemic chronic inflammation, and enhance metabolic efficiency (Yang et al., 2014; Huang et al., 2022). Erchen decoction (ECD) demonstrates notable efficacy in lowering body mass and blood triglyceride concentrations; enhancing gut microbial diversity; and boosting the proportional presence of *Lactobacillus*, *Bifidobacterium*, and *Butyricicoccu*s; while diminishing the abundances of *Bacteroides*, *Parabacteroides*, and *Sediminibacterium* (Matthew et al., 2021; Lulu et al., 2023). Subsequent research indicated that ECD facilitated the restoration of compromised membrane potential, and rectified lipid metabolic abnormalities in mice with obesity (Sergio et al., 2018). Dachaihu decoction (DCHD) can notably improve the reduction in the mitochondrial membrane potential and swelling, activate key factors of mitochondrial synthesis, increase mitochondrial function, and balance the abnormalities of energy metabolism in obese rats (Li L. et al., 2021). Furthermore, DCHD enhanced the intestinal microbiota by restoring *Lactobacillus*, *Akkermansia*, and *Bifidobacterium* levels and elevating the proportion of *Bacteroidetes* to *Firmicutes*, thus contributing to additional weight reduction (Hussain et al., 2016). Gegen Qinlian decoction (GQD) can decrease the production of LPS, and improve abnormalities in lipid metabolism (Xiong, 2020). Moreover, GQD is capable of suppressing IL-6 expression, alleviating obesity-related inflammation, and modulating energy metabolism through stimulating WAT browning and enhancing caloric expenditure (Zhang X. et al., 2021). Wu-Mei-Wan (WMW) can reduce the body weight of obese mice and decrease the ratio of *Firmicutes* to *Bacteroidetes*, thereby adjusting the gut microbiota structure (Nie et al., 2021b). Additionally, WMW treatment resulted in a reduction of white adipocytes, a diminution in lipid droplet quantity, an upregulation of UCP1 expression, an augmentation of mitochondrial count in BAT, and an elevation in energy consumption (Wu et al., 2020).

**TABLE 3:** TCM formulas for the treatment of obesity.

TCM formulas	Composition of the formula	Extraction	Types of study	Experiment object	Dosage	Intervention mode	Modeling methods	Effects on gut Microbiota–Mitochondrial crosstalk	Mechanisms	Metabolic outcomes	References
Shen Ling Bai Zhu San	Panax ginseng C.A. Mey. [Araliaceae; Ginseng Radix et Rhizoma], Atractylodes macrocephala Koidz. [Asteraceae; Atractylodis macrocephalae rhizoma],Poria cocos (Schw.) Wolf [Polyporaceae; Poria],Dioscorea oppositifolia L. [Dioscoreaceae; Dioscoreae rhizoma], *Nelumbo nucifera* Gaertn. [Nelumbonaceae; Nelumbinis semen],Lablab purpureus subsp. purpureus [Fabaceae; Lablabi semen album], Coix lacryma-jobi var. ma-yuen (Rom.Caill.) Stapf [Poaceae; Coicis semen], Wurfbainia villosa (Lour.) Škorničk. and A.D.Poulsen [Zingiberaceae; Amomi fructus], Platycodon grandiflorus (Jacq.) A.DC. [Campanulaceae; Platycodonis radix], Glycyrrhiza uralensis Fisch. ex DC. [Fabaceae; Glycyrrhizae radix et rhizoma]	Mix in a ratio of 5:5:5:5:3:4:3:2:2:3 g in sequence	*In vivo*	Male C57BL/6J mice	21.8 g/kg	Gavage 6 weeks	High-fat diet	↑ *Bifidobacterium*, *Parvibacter*, ↓ *Erysipelatoclostridium*, *Lachnoclostridium*	↓TPH1, 5-HT, HTR2A; ↓IL-6, IL-1β, TNF-α, MCP-1, IL-18	↓ Body weight, ↓ Hepatic steatosis, ↓ Inflammation, ↑ Insulin sensitivity	Chen et al. (2024)
Erchen decoction	Pinellia ternata (Thunb.) Makino [Araceae; Pinelliae Rhizoma],Citrus reticulata Blanco [Rutaceae; Citrus reticulatae pericarpium],Poria cocos (Schw.) Wolf [Polyporaceae; Poria],Glycyrrhiza uralensis Fisch. ex DC. [Fabaceae; Glycyrrhizae radix et rhizoma]	Mixed in a ratio of 15:15:9:4.5 g in sequence. They were decocted twice, and the two decoctions were combined and concentrated to 50 mL	*In vivo*	Male C57BL/7J mice	8.7 g/kg	Gavage 4 weeks	High-fat diet	↑ATP, ↑ Mitochondrial respiration,↑ Mitochondrial membrane potential, ↓ Hepatic lipid accumulation	↓mTORC1, S6K, SREBP1,↑CAV1; ↓TG,TC; ↓IL-6, IL-1β, TNF-α, MCP-1, IL-18	↓ Fasting blood glucose, ↓ Insulin levels, ↓ HOMA-IR	Ding et al. (2024)
		Mixed in a ratio of 15:15:9:4.5 g in sequence. They were soaked in distilled water (1:8, w/v) for 2 h, then boiled at high heat and simmered for 30 min. The extraction was repeated twice, and the filtrates were combined and concentrated to final crude drug concentrations	*In vivo*	Male Zucker Diabetic Fatty rats	2.28, 4.57, 9.14 g/kg	Gavage 4 weeks	High-fat diet	↓ *Prevotella*, *Blautia*, *Ruminococcus*, *Holdemania*, ↑ *Akkermansia*	↑p-IRS1/IRS1 and p-AKT/AKT,↓ p-PKA/PKA and p-HSL/HSL	↓ Body weight, ↓ Fat accumulation, ↓ Inflammation, ↑ Insulin sensitivity, ↓ Circulating free fatty acids	Zhao et al. (2021b)
Dachaihu Decoction	Bupleurum chinense DC. [Apiaceae; Bupleuri Radix],Scutellaria baicalensis Georgi [Lamiaceae; Scutellariae radix],Citrus × aurantium L. [Rutaceae; Aurantii Fructus Immaturus],Paeonia lactiflora Pall. [Paeoniaceae; Paeoniae Radix Alba],Pinellia ternata (Thunb.) Makino [Araceae; Pinelliae Rhizoma],Rheum palmatum L. [Polygonaceae; Rhei Radix et Rhizoma],Zingiber officinale Roscoe [Zingiberaceae; Zingiberis Rhizoma Recens],Ziziphus jujuba Mill. [Rhamnaceae; Jujubae Fructus]	Mixed in a ratio of 15:9:9:9:9:6:15:12 g in sequence	*In vivo*	Male Sprague-Dawley rats	4.25, 8.5, 17 g/kg	Gavage 8 weeks	High-fat diet	↑ Mitochondrial membrane potential, ↓ Mitochondrial swelling and cristae damage,↑ ATP	CREB/PGC-1α Pathway Activation	↓ Hepatic triglycerides, ↓ Total cholesterol,↓ Fasting blood glucose, ↓ Insulin levels	Li et al. (2021a)
Gegen Qinlian Decoction	Pueraria montana var. lobata (Willd.) Maesen and S.M.Almeida ex Sanjappa and Predeep [Fabaceae; Puerariae lobatae radix],Scutellaria baicalensis Georgi [Lamiaceae; Scutellariae radix],Glycyrrhiza uralensis Fisch. ex DC. [Fabaceae; Glycyrrhizae radix et rhizoma],Coptis chinensis Franch. [Ranunculaceae; Coptidis rhizoma]	Mixed in a ratio of 15:9:6:9 g in sequence. They were soaked (1:4, w/v) for 30 min, then boiled for 1 h, followed by a second extraction (1:3, w/v) for 40 min. The filtrates were combined, and concentrated	*In vivo*	Male C57BL/6J mice	15, 45 g/kg	Gavage 8 weeks	High-fat diet	↑ Oxygen consumption, ↑ Mitochondrial UCP1	↑ UCP1 in adipose tissue, ↓ Lipogenic genes	↓ Body weight, ↓ Adipose tissue mass, ↓ Serum triglycerides and cholesterol, ↓ Hepatic lipid accumulation, ↑ Insulin sensitivity	Wang and Hu (2021)
			*In vivo*	Male C57BL/6J mice	12.48, 24.96 g/kg	Gavage 4 weeks	High-fat diet	↓ *Firmicutes/Bacteroidetes* ratio, ↑ *Bacteroidetes*, ↓ *Firmicutes*, ↑ *Verrucomicrobia*	↓ Hepatic triglyceride (TG) and total cholesterol (TC) levels; ↓ Pro-inflammatory cytokines (TNF-α, IL-6, IL-1β)	↓ Body weight, ↓ Hepatic triglycerides and cholesterol, ↓ Fasting blood glucose, ↓ Insulin resistance	Zhang and Zhong (2020)
Wu-Mei-Wan	Prunus mume (Siebold) Siebold and Zucc. [Rosaceae; Mume Fructus],Asarum heterotropoides F.Schmidt [Aristolochiaceae; Asari Radix et Rhizoma],Zingiber officinale Roscoe [Zingiberaceae; Zingiberis Rhizoma Recens],Aconitum carmichaelii Debeaux [Ranunculaceae; Aconiti Lateralis Radix Praeparata],Zanthoxylum bungeanum Maxim. [Rutaceae; Zanthoxyli Pericarpium],Neolitsea cassia (L.) Kosterm. [Lauraceae; Cinnamomi Ramulus],Coptis chinensis Franch. [Ranunculaceae; Coptidis rhizoma],Phellodendron chinense C.K.Schneid. [Rutaceae; Phellodendri Chinensis Cortex],Panax ginseng C.A. Mey. [Araliaceae; Ginseng Radix et Rhizoma],Angelica sinensis (Oliv.) Diels [Apiaceae; Angelicae Sinensis Radix]	Mixed in a ratio of 19.2: 7.2:12:7.2:4.8:7.2:19.2:7.2:7.2:4.8 g in sequence. They were soaked in 500 mL water for 1 h, then boiled for 2 h, with then concentrated	*In vivo*	Male C57BL/6J mice	0.48, 0.96, 1.92 g/mL	Gavage 4 weeks	High-fat diet	↑ Mitochondrial DNA content in BAT, ↑ Thermogenesis-related genes (UCP1, PGC-1α, COX III, CPT1β, CIDEA, ATPsyn, FATP1, MCAD)	↓ TLR3/IL-6/JAK1/STAT3 pathway,↑ BMP7/Smad1/5/9 pathway	↓ Body weight, ↓ White adipose tissue mass, ↑ Brown adipose tissue activity, ↑ Energy expenditure, ↓ Serum triglycerides and total cholestero	Wu et al. (2020)
		Mixed in a ratio of 24: 9:15:9:6:9:24:9:9:6 g in sequence	*In vivo*	Male C57BL/6J mice	4800 mg/kg	Gavage 4 weeks	High-fat diet	↑ *Bacteroidetes*, *Parabacteroides goldsteinii*, *Akkermansia muciniphila*, ↓ *Firmicutes*, *Blautia, Lactobacillus*	↓ Serum triglyceride and total cholesterol,↓ Pro-inflammatory cytokines (TNF-α, IL-6, IL-1β),↑ Short-chain fatty acids	↓ Body weight, ↓ White adipose tissue mass, ↓ Inflammation, ↓ Serum triglycerides and total cholesterol	Nie et al. (2021a)

TCM formulas and metabolites of botanical drugs which are guided by TCM theory targets the bidirectional communication between the gut microbiota and mitochondria, offering a multilevel intervention strategy for obesity treatment. However, further research is needed to elucidate these therapies’ long-term effects and potential side effects across different individuals and clinical settings.

## 7 Conclusion and prospects

The intricate crosstalk between the gut microbiota and mitochondria is crucial in obesity and related metabolic disorders. The gut microbiota and its metabolites substantially affect host adipogenesis, modulating mitochondrial energy production and metabolism with reciprocal impacts. This bidirectional interaction highlights the gut microbiota’s capacity to influence the host’s metabolic wellbeing, while also indicating that mitochondria, essential cellular structures responsive to internal and external stimuli, reciprocally affect the gut microbiota by modulating mucosal immunity and intestinal barrier. Consequently, this reciprocal relationship between mitochondria and the gut microbiota holds promise for addressing obesity. Given the evolutionary origin of mitochondria as primordial bacterial symbionts, the intricate connection between these organelles and bacteria is understandable. Emerging evidence suggests that TCM formulas and metabolites of botanical drugs could provide novel insights into restoring this gut microbiota–mitochondria axis. This review consolidates findings supporting a connection between gut microbiota imbalances in obesity and mitochondria, offering potential pathways for investigating innovative treatment strategies for obesity.

However, TCM emphasizes individualized treatment, with personalized prescriptions based on the patient’s pathological state of obesity and its comorbidities according to the different stages of development. Most current studies rely on simple obesity or simple metabolic syndrome models, which fail to effectively simulate the complex pathology of obese patients, especially when multiple metabolic complications coexist, and the interactions between the gut microbiota and mitochondrial function may show more complex dynamics. In addition, although studies have proposed that the interaction between gut microbiota and mitochondria plays a key role in the combined effects of obesity onset and progression. Limited comparability of data obtained from different experimental approaches further constrains the integrative interpretation of these findings. Therefore, although formulas and metabolites of botanical drugs have shown potential to alleviate obesity by modulating mitochondria and gut microbiota, their specific mechanisms through gut microbiota-mitochondria interactions still need to be further validated.

Future research should prioritize the development of multifactorial obesity models that more accurately reflect human pathological characteristics, particularly in cases where obesity is accompanied by metabolic comorbidities such as type 2 diabetes, metabolic dysfunction-associated fatty liver disease, and cardiovascular diseases. These models will enable a more precise simulation of clinical conditions, enhancing the translational relevance of research findings and facilitating the optimization of TCM formulations and metabolites of botanical drugs for different pathological states. Furthermore, integrating organoid technology and tissue engineering to establish multi-organ co-culture systems will allow for a more physiologically relevant simulation of systemic interactions, providing a robust platform for investigating the gut microbiota–mitochondria axis and its potential therapeutic mechanisms. Additionally, the combination of synthetic biology and intelligent compounding technologies offers opportunities to optimize drug delivery strategies. Approaches such as nanocarrier systems or pre-drug design can enhance targeting efficiency while reducing off-target effects, thereby providing a strong scientific foundation for the precise intervention of TCM formulations and the metabolites of botanical drugs through modulation of the gut microbiota–mitochondrial crosstalk in obesity and metabolic diseases.
